# Tuning of Rashba/Dresselhaus Spin Splittings by Inserting Ultra-Thin InAs Layers at Interfaces in Insulating GaAs/AlGaAs Quantum Wells

**DOI:** 10.1186/s11671-016-1671-7

**Published:** 2016-10-26

**Authors:** Jinling Yu, Xiaolin Zeng, Shuying Cheng, Yonghai Chen, Yu Liu, Yunfeng Lai, Qiao Zheng, Jun Ren

**Affiliations:** 1 Institute of Micro/Nano Devices and Solar Cells, School of Physics and Information Engineering, Fuzhou University, Fuzhou, China; 2 Key Laboratory of Semiconductor Materials Science, Institute of Semiconductors, Chinese Academy of Sciences, and University of Chinese Academy of Sciences, P.O. Box 912, Beijing, 100083 People’s Republic of China; 3 Jiangsu Collaborative Innovation Center of Photovolatic Science and Engineering, Changzhou University, Changzhou, Jiangsu, 213164 China; 4 Department of Physics, State Key Laboratory of Low Dimensional Quantum Physics, Tsinghua University, Beijing, 100084 China

**Keywords:** Spectroscopy of circular photogalvanic effect, Ratio of Rashba/Dresselhaus spin splittings, Rashba- and Dresselhaus-type CPGE, Interface inversion asymmetry, Reflectance difference spectroscopy

## Abstract

The ratio of Rashba and Dresselhaus spin splittings of the (001)-grown GaAs/AlGaAs quantum wells (QWs), investigated by the spin photocurrent spectra induced by circular photogalvanic effect (CPGE) at inter-band excitation, has been effectively tuned by changing the well width of QWs and by inserting a one-monolayer-thick InAs layer at interfaces of GaAs/AlGaAs QWs. Reflectance difference spectroscopy (RDS) is also employed to study the interface asymmetry of the QWs, whose results are in good agreement with that obtained by CPGE measurements. It is demonstrated that the inserted ultra-thin InAs layers will not only introduce structure inversion asymmetry (SIA), but also result in additional interface inversion asymmetry (IIA), whose effect is much stronger in QWs with smaller well width. It is also found that the inserted InAs layer brings in larger SIA than IIA. The origins of the additional SIA and IIA introduced by the inserted ultra-thin InAs layer have been discussed.

## Background

Nowadays, spintronics has attracted enormous research interest since it promises to revolutionize electronics and computing by making explicit use of the electron’s spin in addition to its charge [1–3]. The spin-orbit coupling (SOC) provides a mechanism for the generation and manipulation of spins solely through electric fields [4]. SOC can be divided into two types, one is Dresselhaus term induced by the bulk inversion asymmetry (BIA)[5] and by interface inversion asymmetry (IIA), and the other is Rashba term induced by the structure inversion asymmetry (SIA)[6–8]. IIA yields BIA-like terms in the effective Hamiltonian for electrons [9, 10], which make it difficult to separate these two terms experimentally. As a result, there are few works reporting the experimental investigations of IIA-induced SOC. CPGE is recently emerging as an effective experimental tool to measure SOC in low-dimensional semiconductor system [10–12], which can separate Rashba- and Dresselhaus-type SOC of the quantum wells (QWs) belonging to zinc-blende structure by adopting different optical geometries [13, 14]. Besides, reflectance difference spectroscopy (RDS) is a powerful tool to investigate the interface asymmetry, which can measure the tiny optical anisotropy (OA) induced by interface asymmetry [15]. Therefore, it may be a good idea to employ these two methods to study the IIA-induced SOC.

It has been recognized that Rashba and Dresselhaus SOC can interfere with each other and result in an anisotropy of spin splitting [10]. If these two terms have equal strength with each other, the spin splitting in certain *k*-space directions will vanish. This can lead to some new macroscopic effects, such as the lack of Shubnikov-de Haas beating or the disappearance of spin relaxation in specific crystallographic directions [16]. These effects can be employed for a nonballistic spin-field effect transistor [17]. Therefore, effectively tuning the ratio of Rashba and Dresselhaus SOC (RD ratio) is of great importance for designing new kinds of spintronics devices. Ganichev et. al. tuned the RD ratio by shifting the *δ*-doping plane from one side of the quantum well to the other and measured the RD ratio by circular photogalvanic effect (CPGE) [14]. In our previous work, the RD ratio is tuned by changing the temperature [18].

In this paper, we tune the RD ratio by changing the well widths and by inserting an ultra-thin InAs layer with a thickness of one monolayer (ML) at interfaces of GaAs/AlGaAs multiple quantum-wells (MQWs), which is measured by CPGE spectra at inter-band excitation. We also investigate the IIA-induced SOC by comparing the CPGE and RDS spectra. It is found that the RD ratio can be effectively tuned by changing the well width and by the inserted InAs layer. Besides, we also find that the inserted ultra-thin InAs layer will not only introduce SIA, but also result in additional IIA, whose effect is much stronger for QWs with smaller well width.

## Methods

**Fig. 1:**
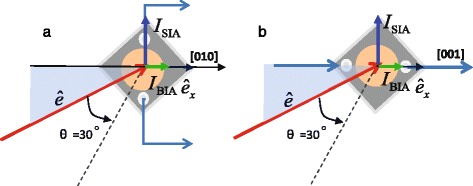
Geometries used to measure the CPGE current. **a** and **b** are used to measure the CPGE current induced by Rashba- and Dresselhaus-type SOC, respectively

We study four undoped GaAs/Al _0.3_ *Ga*_0.7_As MQWs samples grown on (001) SI-GaAs substrates by molecular beam epitaxy (MBE). Sample A and B contain 20 periods of 7 and 3 nm quantum wells, respectively. Sample C and D have the same structure with that of sample A and B, respectively, except that an ultra-thin InAs layer with a thickness of 1 ML is inserted at one of the interfaces of each GaAs/Al _0.3_ *Ga*_0.7_As QWs, i.e., forming Al _0.3_ *Ga*_0.7_As/InAs/GaAs/Al _0.3_ *Ga*_0.7_As structures. The InAs layer is grown at 500 °C. In order to prevent the volatilization of InAs when growing GaAs layer at a higher temperature of 580 °C, a GaAs layer of 1 nm is grown on top of the InAs layer, followed by heating up of the substrate to 580 °C to grow the rest GaAs and Al _0.3_ *Ga*_0.7_As layers. After the growth of 20 periods of QWs, the samples are capped by 100 nm Al _0.3_ *Ga*_0.7_As and 20 nm GaAs layer. All of the four samples are of high resistance without lighting at room temperature, indicating the high purity of the samples. The 2D densities of the photo-induced carriers corresponding to the transition of 1H1E (the first valence subband of heavy holes to the first conduction subband of electrons) for the four samples are all estimated to be about 10^9^ cm ^−2^ under a radiation of a laser with a power of 60 mW. For CPGE measurements, the samples are cleaved along [110] and [1$\bar {1}$0] directions into squares of 4 ×4 mm^2^. Then one pair of ohmic contacts with 3 mm apart along [100] direction is made by indium deposition and annealed at about 420 °C in nitrogen atmosphere, as shown in Fig. 1 a, b.

A mode-locked Ti-sapphire laser with a repetition rate of 80 MHz is used as the radiation source. The full width at half maximum (FWHM) of the laser pulse is about 7 nm. The light then goes through a polarizer and a photoelastic modulator (PEM) to become a periodically oscillating polarization between right- (*σ* ^−^) and left- (*σ* ^+^) hand circularly polarized light. Finally, a Gaussian profile light spot with a diameter of about 2.5 mm irradiates at the central line between two electrodes with a power of about 60 mW at 840 nm. In order to extract the common photocurrent *I*_0_ under DC bias, a chopper with a frequency of 220 Hz and a lock-in amplifier are used. The photogalvanic current is measured in the unbiased structure via a preamplifier and then is recorded by the lock-in amplifier in phase with the PEM. The wavelength of the light ranges from 750 to 870 nm.

Due to the large FWHM of the laser pulse of the Ti-sapphire laser, the transitions of 1H1E and 1L1E (the first valence subband of light holes to the first conduction subband of electrons) of the QWs can not be clearly distinguished in the common photocurrent spectra, denoted as *I*_0_. Thus, we replace the Ti-sapphire laser with a 250-W tungsten lamp combined with a monochromator, which has a spectral resolution of 1 nm, to measure the common photocurrent spectra, denoted as *I*_1_. The DC bias is adopted to be 3 V.

Since the Rashba and Dresselhaus SOC contribute differently to the CPGE for different crystallographic directions, we can separate the spin splitting induced by Rashba and Dresselhaus SOC according to the method proposed in [13] and [14]. Therefore, using the geometry shown in Fig. 1 a, b, we can obtain the CPGE current induced by Rashba and Dresselhaus spin splitting, respectively.

In order to investigate the interface asymmetry of the four samples, we perform RDS measurements at 77 K. RDS can precisely measure the reflectance difference between [110] and [1$\bar {1}$0] directions, i.e., $\Delta r/r=2(r_{110}-r_{1\bar {1}0})/(r_{110}+r_{1\bar {1}0})$, resulted from interface asymmetry of the QWs. Here, *r*_110_ denotes the reflectance coefficient of the sample when the incident light is polarized along [110] direction. The RDS setup is the same as that used in [19].

## Results and discussion

**Fig. 2:**
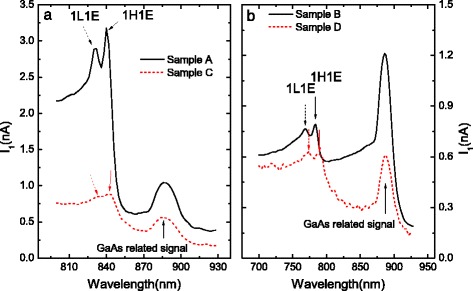
Common photocurrent *I*_1_ under a DC bias of 3 V for **a** samples A, C and **b** sample B, D, respectively. The *arrows* indicate the energy positions of the transition 1H1E, 1L1E, and that related to GaAs bulk material

**Fig. 3:**
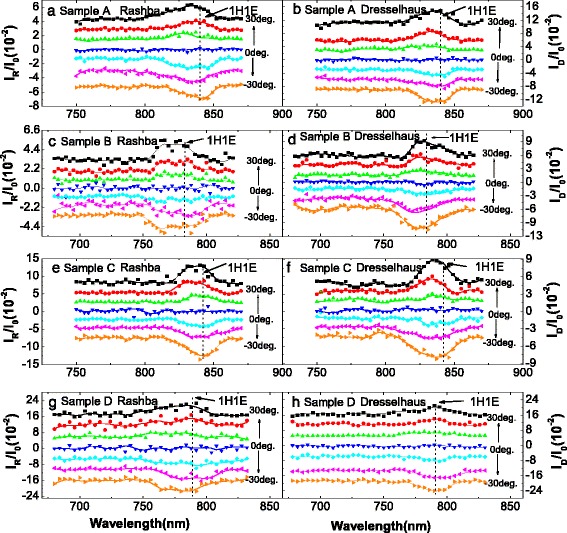
CPGE spectra for samples A–D induced by Rashba- and Dresselhaus-type SOC normalized by the common photocurrent *I*_0_ at the angles of incidence from −30 to 30 °C with a step of 10 °C. **a**, **c**, **e** and **g** are the CPGE current induced by Rashba-type SOC, and **b**, **d**, **f** and **h** are those induced by Dresselhaus-type SOC. All of the spectra are shifted vertically for clarity. The *vertical dash lines* indicate the energy position of the transition of 1H1E. The *solid lines* are guides for eyes

**Fig. 4:**
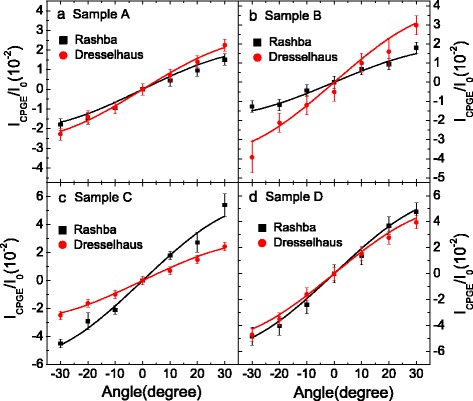
Angular dependence of the normalized CPGE current for sample A–D induced by Rashba- and Dresselhaus-type SOC for the transition of 1H1E, respectively. The *squares* and *circles* are experiential results and the *solid lines* are the fitting results according to Eq. (1)

Figure 2 shows the common photocurrent *I*_1_ under a DC bias of 3 V for samples A, B, C, and D. The signal related to the transitions of 1H1E and 1L1E can be clearly distinguished, which are marked by downward arrows. It can be seen that the transition energies of 1H1E and 1L1E in sample C (D) shift a little to lower energy compared to that of sample A (B), due to the perturbation of the inserted InAs layer. Figure 3 shows the CPGE spectra normalized by the common current *I*_0_ for sample A, B, C, and D induced by Rashba- and Dresselhaus-type SOC, respectively, at different angles of incidence. The Rashba-type spectra in Fig. 3 are measured under the geometry shown in Fig. 1 a, and the Dresselhaus-type spectra are obtained under the geometry of Fig. 1 b. In the CPGE spectra, it is difficult to make a distinction between the signal related to 1H1E and that related to 1L1E due to the large FWHM of the Ti-sapphire laser. In order to distinguish the CPGE signal related to 1H1E and 1L1E, we should turn to the common photocurrent spectra *I*_1_ shown in Fig. 2 to locate the energy positions of 1H1E and 1L1E. Since the CPGE signal associated with 1H1E and 1L1E shows similar behaviors, we only focus on that related to 1H1E in the following discussion. So, we normalized the CPGE spectra by the common current *I*_0_ corresponding to the transition of 1H1E, whose energy positions can be clearly observed in the common photocurrent spectra *I*_1_ shown in Fig. 2 and are indicated by vertical dashed lines in Fig. 3. Thus, the influences of the carrier mobility and carrier density in different samples can be eliminated, which will allow us to compare CPGE current in different samples. It can be seen that for the investigated four samples, the Rashba-type CPGE spectra shows similar lineshape with that of Dresselhaus-type, which are not consistent with the theoretical predictions reported in [20]. The discrepancies can be mainly attributed to the excitonic effect, which may play a dominant role in the inter-band resonance excitation CPGE spectra of the insulating QWs [18, 21]. Figure 4 shows the incident angle dependence of the magnitude of the Rashba- and Dresselhaus-type CPGE current corresponding to the transition of 1H1E for the four samples. The solid lines in the figure are the fitting results according to the following equation

1 $$ {}I_{\lambda}/I_{0}\,=\,\frac{A\sin\theta \cos^{2} \theta}{n(\cos\theta\,+\,\sqrt{n^{2}\,-\,\sin^{2}\theta})(n^{2}\cos\theta\,+\,\sqrt{n^{2}\,-\,\sin^{2}\sin\theta})}.  $$

Here, *n* is the refractive index, and *λ*=R or D corresponds to the CPGE current induced by the Rashba- or Dresslhaus-type SOC, respectively. If *λ*= R, *A*=*a* *α* *P*_circ_; if *λ*= D, *A*=*a* *β* *P*_circ_, where *P*_circ_ is the degree of circular polarization given by $\phantom {\dot {i}\!}P_{\text {circ}}=(I_{\sigma ^{+}}-I_{\sigma ^{-}})/(I_{\sigma ^{+}}+I_{\sigma ^{-}})$, and *a* is a constant determined by the optical selection rules and by the momentum relaxation time. It is worth mentioning that, (i) we only take the *k*-linear Hamiltonian in the conduction band in the derivation of Eq. (1), because the cubic in *k* Dresselhaus terms of conduction band are usually unimportant in GaAs QWs, which may play an important role in narrow band materials and highly doped QWs as well as at high temperature [22]; (ii) *α* (or *β*) should be the effective Rashba (or Dresselhaus) parameter proportional to the effective strength of Rahsba (or Dresselhaus) spin splitting in the QWs, since the CPGE current induced by inter-band excitation is determined by the spin splitting both in conduction and valence bands. What is more, the contribution of the holes to the CPGE may be comparable or even larger than that of electrons in the samples with InAs layers inserted at the interfaces of the QWs, because there is large SOC in the valence bands induced by the heavy-light hole interface mixing in such kinds of samples [23, 24], and it will result in much larger momentum relaxation time of holes compared with that of electrons; (iii) although interface mixing makes a significant contribution both to *k*-linear and *k* ^3^ terms in the valence band effective Hamiltonian, the *k*-linear term plays a dominant role at small *k*, which is the case of our samples where the excitonic effect makes a major contribution to the CPGE current. Therefore, Eq. (1) still holds even the SOC of the valence bands is further taken into account.

In the fittings of Eq. (1), the refractive index *n* of the QWs material is adopted to be 3.55 according to [25], and the parameter *A* is fitted to be 1000 ±77 and 1274 ±45 for the normalized Rashba- and Dresselhaus-type CPGE current in sample A, respectively. Therefore, we can obtain the RD ratio *α*/*β*=(1000±77)/(1274±45)=0.78±0.08. Similarly, the parameter *A* in the normalized Rashba-type CPGE current *I*_R_/ *I*_0_ of the transition 1H1E in samples B, C, and D are 891 ±60, 2756 ±205, 2945 ±112, respectively, and that related to the normalized Dresselhaus-type CPGE current *I*_D_/ *I*_0_ in samples B, C, and D are 1849 ±162, 1389 ±50, 2554 ±104, respectively, as shown in Table I. Thus, we can obtain the RD ratios for the transition of 1H1E in sample B, C and D are 0.48 ±0.08, 1.98 ±0.23 and 1.15 ±0.10, respectively, which are also shown in Table I. It can be seen that the RD ratio of the QWs can be effectively tuned by changing the well width of the QWs and by the inserted ultra-thin InAs layers.

Denoting the Rashba and Dresselhaus coefficent for sample *i* (*i*=A, B, C or D) as *α*_*i*_ and *β*_*i*_, and marking the parameter *A* deduced from Rashba- and Dresselhaus-type CPGE current of sample *i* (*i*=A, B, C or D) as *A*_R*i*_ and *A*_D*i*_, respectively, we find that *A*_RA_ almost equals to *A*_RB_ within experimental errors, indicating that the Rashba SOC in sample B nearly equals to that in sample A, i.e., *α*_A_≃*α*_B_. This is because in the symmetric GaAs/AlGaAs QWs, the Rashba SOC mainly stems from the built-in electric field, which is due to the residual doping. Since sample A and sample B are grown under the same conditions and they have the same structure except for the well width of the QW (7 versus 3 nm), the residual doping and the consequent built-in field will be approximately equal, resulting in similar value of Rashba-type SOC. However, the ratio between *A*_DB_ and *A*_DA_ is *z*=*A*_DB_/*A*_DA_=1.63±0.47, indicating a larger Dresselhaus-type SOC in sample B. This observation is in good agreement with that observed in Ref. [26] within experimental error, which reported that the ratio of Dresselhaus-type SOC between the sample with 3 and 7 nm well width is about 1.57. This phenomenon can be attributed to the following two reasons: firstly, as pointed out in Ref. [22] and [26], the smaller the well width is, the larger confinement it provides for the carriers in the QW, leading to larger Dresselhaus spin splitting; secondly, as evident by the OA measurements of the four samples shown below, samples with smaller well width have larger IIA resulting in larger Dresselhaus spin splitting. We also note that *A*_RC_ nearly equals to *A*_RD_ within experimental error, implying the same strength of Rashba-type SOC in the two samples. This is due to the fact that they are grown under the same conditions and they have the same structure except for the well width of the QW. One can see that, *A*_RC_ is much larger than *A*_RA_ with a ratio of *Y*=*A*_RC_/*A*_RA_=2.76±0.45, showing that the inserted ultra-thin InAs layer introduces additional SIA into the QWs. This is because the band gap of InAs (about 0.35 eV) is much smaller than that of GaAs (about 1.42 eV), which results in significant asymmetry along [001] direction. It is interesting to note that the Dresselhaus-type SOC in sample C is a little larger than that in sample A, since *X*=*A*_DC_/*A*_DA_=1.09±0.08. This indicates that the inserted InAs layer also bring in additional Dresselhaus-type SOC, which, probably, results from interface inversion asymmetry (IIA), since the ultra thin InAs layer will result in different chemical bonds at the two interfaces of the QWs [24], i.e., the left interface involves Al-As and Ga-As bonds, in a ratio of 0.35 to 0.65, lying in the ($\bar {1}$10) plane and Ga-As bonds lying in the perpendicular (110) plane, while the right interface consists of Ga-As bonds in the ($\bar {1}$10) plane and In-As bonds in the (110) plane. The additional IIA introduced by InAs layer is also evident from the larger OA intensity in sample C compared with that of sample A measured by RDS spectra shown below. Because IIA yields BIA-like terms in the effective Hamiltonian, IIA will introduce Dresselhaus-type SOC. As a result, the spin splittings both in the valence and conduction band will be enhanced, and the effect on the spin splitting in the valence band is much larger than that in the conduction band [7, 23]. The IIA will lead to the *k*-linear Hamiltonian the same as that derived from the BIA for electrons [7], but results in both *k*-linear terms, which play a leading role at small *k*, and *k* ^3^ terms for holes [23]. Since the excitonic effect plays a dominant role in the inter-band excited CPGE spectra of the insulating GaAs/AlGaAs QWs [21], the CPGE current is mainly contributed by the carriers at small *k*. Therefore, the *k* ^3^ terms induced by IIA in the valence band can be neglected. Besides, we also note that the ratio of *A*_DD_ to *A*_DB_ is larger than that of *A*_DC_ to *A*_DA_, i.e., *z*_2_=*A*_DD_/*A*_DB_=1.38±0.19>*X*, suggesting that the additional IIA introduced by the inserted InAs layer is larger in QWs with smaller well width. This phenomenon can be owing to stronger hole-mixing effect at smaller well width evident from the enhanced OA intensity induced by IIA in the QWs with smaller well width as shown below. The relations of *Y*>*X* and *Y*>*z*_2_ indicate that the inserted ultra-thin InAs layer will introduce larger SIA than IIA for the investigated 7 and 3 nm GaAs/AlGaAs QWs due to the striking asymmetry along the growth direction of the QWs as induced by the remarkable difference between the band gap of InAs and GaAs.

**Fig. 5:**
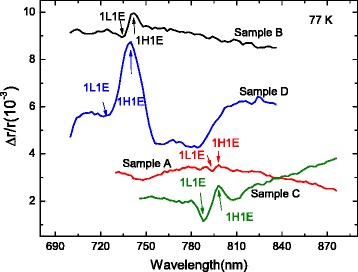
Reflectance difference spectra of the four samples measured at 77 K. The spectra are shifted vertically for clarity. The *arrows* indicate the energy positions of 1H1E and 1L1E

The interface asymmetry can be evident from the OA in the interface planes as a consequence of the mixing effect of the heavy- and light-hole induced by interface asymmetry. In order to investigate the interface asymmetry of the four samples, we perform RDS measurements at 77 K and the results are shown in Fig. 5. All of the spectra are shifted vertically for clarity. The arrows indicates the energy positions of the transitions of 1H1E and 1L1E. One can see that the inserted InAs layer will make the transitions of 1H1E and 1L1E shift to lower energy slightly, which are also observed in the common photocurrent spectra *I*_1_. It can be seen that the transitions of 1H1E and 1L1E have opposite OA, since the value of *Δ* *r*/*r* reflects the OA intensity in the two interfaces of the QW. The OA intensity of 1H1E are 0.13 ±0.05, 0.60 ±0.05, 0.75 ±0.05 and 3.00 ±0.05 for sample A, B, C and D, respectively, as shown in Table 1. The OA in sample A and B mainly comes from anisotropic interface defects or anisotropic atomic segregation at AlAs-on-GaAs interface [15], while that in sample C and D mainly stems from the anisotropic chemical bonds at the interface. It can be clearly seen that the OA intensity in sample C is about six times larger than that in sample A, indicating larger interface asymmetry induced by the inserted InAs layer, which also support the results of CPGE measurements. Besides, the OA intensity in sample D (B) is larger than that in sample C (A), suggesting that the interface asymmetry is larger at smaller well width, due to the larger ratio of the number of interface atoms to that of bulk atoms for smaller well width [24]. The increase of OA intensity from sample C to sample D (2.25 ×10^−3^) is larger than that from sample A to B (0.47 ×10^−3^), implying that the additional interface asymmetry introduced by the inserted InAs layer is also larger at smaller well width. This observation consists again with that is found in CPGE measurements.

**Table 1:** The fitted parameter *A* of the normalized Rashba- and Dresselhaus-type CPGE current, the ratio of Rashba and Dresselhaus SOC (RD ratio) and the OA intensity corresponding to the transition of 1H1E for the four samples

	Sample A	Sample B	Sample C	Sample D
Parameter *A* in *I*_R_/*I*_0_	1000 ±77	891 ±60	2756 ±205	2945 ±112
Parameter *A* in *I*_D_/*I*_0_	1274 ±45	1849 ±162	1389 ±50	2554 ±104
RD ratio	0.78 ±0.08	0.48 ±0.08	1.98 ±0.23	1.15 ±0.10
*Δ* *r*/*r* (10 ^−3^)	0.13 ±0.05	0.60 ±0.05	0.75 ±0.05	3.00 ±0.05

## Conclusions

In conclusion, the influence of the well width of the QWs on the CPGE spectra induced by Rashba and Dresselhaus SOC at inter-band excitation have been experimentally investigated in (001)-grown GaAs/AlGaAs QWs. Ultra-thin InAs layers with a thickness of 1 ML have been inserted at the interfaces of GaAs/AlGaAs QWs to tune the asymmetry of the QWs. The interface asymmetry of the QWs with and without InAs inserted layers are also investigated by RDS at 77 K. The results obtained by the CPGE and RDS measurements are consistent with each other. We find that the RD ratio of the QWs can be effectively tuned by the well width and by the inserted InAs layer. It is also found that the inserted ultra-thin InAs layer will not only introduce SIA, but also result in additional IIA, and the SIA is larger than the IIA. Besides, both evident from CPGE and RDS measurements, the IIA are much stronger in QWs with smaller well width.
